# Medicines—Aims and Scope Updates

**DOI:** 10.3390/medicines11050011

**Published:** 2024-05-14

**Authors:** Hiroshi Sakagami

**Affiliations:** Meikai University Research Institute of Odontology (M-RIO), 1-1 Keyakidai, Sakado 350-0283, Japan; sakagami@dent.meikai.ac.jp

The journal *Medicines* (ISSN 2305-6320) was launched in 2014. Since then, it has had numerous interesting publications covering all areas of medical disciplines and subspecialties. Following the latest feedback from Scopus, we are updating our scope so it is more in alignment with the papers published in our journal.

We aim to better serve the readers and authors by providing more space for publication in the fields of therapeutic agents and advanced drug studies, but not in those of diet and nutrition, biological and stem-cell therapy, radiation and other nonsurgical therapeutic strategies, palliative care and physical therapy, and multimodal therapy.

Following a thorough review of the published papers and developments in the research fields that we address, we concluded that the subjects that fall within the scope of this journal are perhaps too extensive and could benefit from some fine adjustments. More details are presented in Table 1.

For more information about journal *Medicines*, please visit the following link: https://www.mdpi.com/journal/medicines/.

## Figures and Tables

**Table 1:** Changes in the scope of the journal *Medicines*.

Aims (New Version)	Aims (Old Version)
*Medicines* (ISSN 2305-6320) is an international, peer-reviewed, and open-access journal aimed at disseminating cutting-edge research in the field of drug discovery and clinical application. This journal welcomes manuscripts covering a wide range of aspects, including the search for target molecules (e.g., receptors), specificity, side effects, hormesis (bimodal actions), emergence of resistance during long-term administration, potentiation by combinational treatment with more than two drugs, and factors that modify its pharmacological actions (such as sex differences, aging, environmental factors, and physiological fluids). Our aim is to encourage scientists to publish their experimental and theoretical results in as much detail as possible. Full details must be provided when publishing an experiment so that the results can be reproduced.	*Medicines* (ISSN 2305-6320) is an international, peer-reviewed, and open access journal aimed at combining all areas of medical disciplines and subspecialties in one platform. It mainly publishes, but is not limited to, original research papers, case reports, reviews, systematic reviews, brief reports, concepts and opinions on all aspects of basic, clinical, and translational research in relation to human health. The journal will accept manuscripts on medical concepts that include evidence-based medicine and/or alternative medicine. Our aim is to encourage scientists to publish their experimental and theoretical results in as much detail as possible. Full details must be provided when publishing an experiment so that the results can be reproduced. There is, in addition, a unique feature of this journal: in studies on complex mixtures of natural products, the characterization of chemicals using analytical methodologies, such as HPLC, MS, LC–MS, HPLC–MS, and NMR, should be included. Pure identification, chemical, and analytical studies are not enough because in vitro or in vivo experiments that investigate their function are needed. It is expected that the cellular and/or molecular mechanisms of natural compounds or characterized extracts will be analyzed in papers.
**Scope (New Version)**	**Scope (Old Version)**
The following items are suggested (lined up alphabetically): Clinical application of drugs Combination effects of more than two drugs Drug development Hormesis Identification of the target molecule of the drug Modulation factors of drugs (such as aging, sex, microenvironment, and physiological fluids) Overcoming drug tolerance Pharmacodynamics Pharmacokinetics Specificity and adverse effects of drugs	The main subjects covered include but are not limited to following: Acpuncture and herbal medicine Cancer biology and anticancer therapeutics Cardiology and vascular disease Endocrinology and metabolic disorders Gastroenterology & hepatopancreatobiliary medicine Immunologic and inherited diseases Infectious diseases Mental health Nephrology and urology Neurology and neurologic diseases Oral medicine and dentistry Primary care Radiology Obstetrics, gynecology and reproductive medicine Stem cell investigation and regenerative medicine Drug exploration and development

